# A86 COST-EFFECTIVE ANALYSIS OF PRELIMINARY SINGLE-OPERATOR CHOLANGIOSCOPY IN MANAGEMENT OF DIFFICULT STONES

**DOI:** 10.1093/jcag/gwab049.085

**Published:** 2022-02-21

**Authors:** I Sljivic, R Trasolini, F Donnellan

**Affiliations:** 1 Medicine, University of British Columbia, Richmond, BC, Canada; 2 Vancouver General Hospital, Vancouver, BC, Canada

## Abstract

**Background:**

Single-operator peroral cholangioscopy (POC) is a therapeutic modality for difficult biliary stone disease. Given its high success rate and increasing availability, analysis of the economic impact of early POC utilization is critical for clinical decision-making.

**Aims:**

We aim to compare the cost-effectiveness of different first and second-line endoscopic modalities for difficult-to-treat choledocholithiasis.

**Methods:**

A decision tree model with a 1-year time horizon and a hypothetical cohort of 200 patients was used to analyze the cost-effectiveness of POC for first, second and third-line intervention in presumed difficult biliary stones. We adopted the perspective of a Canadian tertiary hospital, omitting complications and recurrence rates associated with ERCP. Effectiveness estimates were obtained from updated meta-analyses. One-way sensitivity analyses and probabilistic sensitivity analyses were also performed to assess how changes in key parameters affected model conclusions.

**Results:**

First and second-line POC achieved comparable clinical efficacy from 95.5% to 96.5% stone clearance. The least expensive strategy is third-line POC (POC-3: $769,151). Performing POC during the second ERCP was marginally more expensive (POC-2: $770,832) but 9% more effective. The strategy of first-line POC incurred the highest hospital expenditures (POC-1: $848,141) but decreased total procedures performed by 13.4% when compared with POC-2. Sensitivity analysis was robust in showing POC-2 as the most optimal approach.

**Conclusions:**

Second-line POC was superior to first and third-line POC for treatment of difficult biliary stones. When based on meta-analysis of non-heterogeneous trials, POC-2 is more cost-effective and cost-efficient. Our study warrants a larger pragmatic effectiveness trial.

**Funding Agencies:**

None

**Figure:**
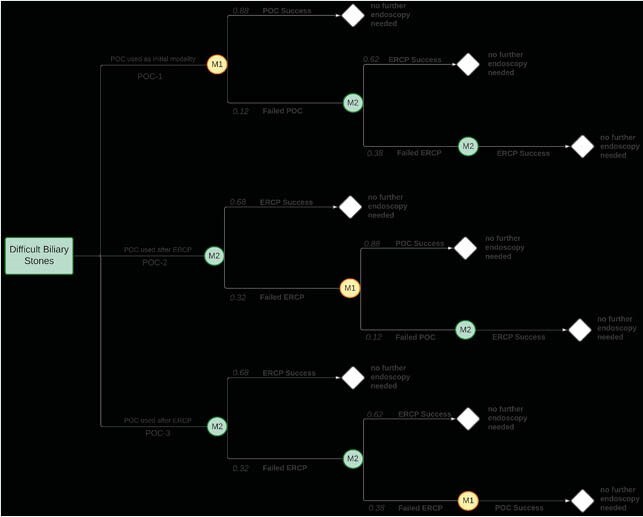
**FIGURE 1**: Management model for treating difficult biliary stones POC, peroral cholangioscopy; ERCP, endoscopic retrograde cholangioscopy. M1 indicates a decision to use ERCP. M2 indicates a decision to use POC. POC-1 – POC used as initial step. POC-2 – POC delayed until after ERCP failure. POC-3 – POC delayed until two failed ERCP attempts.

